# Treatment of Refractory Low Back Pain Using Passive Recharge Burst in Patients Without Options for Corrective Surgery: Findings and Results From the DISTINCT Study, a Prospective Randomized Multicenter Controlled Trial

**DOI:** 10.1016/j.neurom.2023.07.009

**Published:** 2023-08-28

**Authors:** Timothy Deer, Christopher Gilligan, Steven Falowski, Mehul Desai, Julie Pilitsis, Jessica Jameson, Susan Moeschler, Robert Heros, Edward Tavel, Anne Christopher, Denis Patterson, Sayed Wahezi, Jacqueline Weisbein, Ajay Antony, Robert Funk, Mohab Ibrahim, Chi Lim, Derron Wilson, Michael Fishell, Keith Scarfo, David Dickerson, Edward Braun, Patrick Buchanan, Robert M. Levy, Nathan Miller, Jonathan Duncan, Jijun Xu, Kenneth Candido, Scott Kreiner, Marie E. Fahey, James Yue

**Affiliations:** 1 The Spine and Nerve Center of the Virginias, Charleston, WV, USA;; 2 Brigham & Women’s Hospital, Boston, MA, USA;; 3 Center for Interventional Pain & Spine, Lancaster, PA, USA;; 4 International Spine, Pain & Performance Center, Washington, DC, USA;; 5 Florida Atlantic University, FL, USA;; 6 Axis Spine Center, ID, USA;; 7 Mayo Clinic, Rochester, NY, USA;; 8 Spinal Diagnostics, OR, USA;; 9 Clinical Trials of South Carolina, Charleston, SC, USA;; 10 Saint Louis Pain Consultants, Chesterfield, MO, USA;; 11 Nevada Advanced Pain Specialists, Reno, NV, USA;; 12 Montefiore Medical Center—Waters Place, New York, NY, USA;; 13 Napa Valley Orthopedic Medical Group, Napa, CA, USA;; 14 The Orthopaedic Institute, FL, USA;; 15 Indiana Spine Group, IN, USA;; 16 Banner University Medical Center Tucson Campus, Tucson, AZ, USA;; 17 Carolina Orthopaedic & Neurosurgical Associates, SC, USA;; 18 Goodman Campbell Brain & Spine, IN, USA;; 19 Advanced Pain Care, NV, USA;; 20 Rhode Island Hospital, Providence, RI, USA;; 21 Northshore University HealthSystem, Chicago, IL, USA;; 22 Kansas University Medical Center, KS, USA;; 23 Spanish Hills Interventional Pain Specialists, Camarillo, CA, USA;; 24 Anesthesia Pain Care Consultants—Tamarac;; 25 Coastal Pain & Spinal Diagnostics Medical Group, Carlsbad, CA, USA;; 26 Burkhart Research Institute for Orthopaedics, San Antonio, TX, USA;; 27 The Cleveland Clinic Foundation, OH, USA;; 28 Chicago Anesthesia Associates, SC, Chicago, IL, USA;; 29 Barrow Brain & Spine—Ahwatukee, Phoenix, AZ, USA;; 30 Abbott Labs, Austin, TX, USA;; 31 Connecticut Orthopaedics, CT, USA

**Keywords:** composite outcomes, persistent spinal pain syndrome, refractory chronic low back pain, spinal cord stimulation

## Abstract

**Objective:**

Spinal cord stimulation (SCS) is effective for relieving chronic intractable pain conditions. The Dorsal spInal cord STImulatioN vs mediCal management for the Treatment of low back pain study evaluates the effectiveness of SCS compared with conventional medical management (CMM) in the treatment of chronic low back pain in patients who had not undergone and were not candidates for lumbar spine surgery.

**Methods and Materials:**

Patients were randomized to passive recharge burst therapy (*n* = 162) or CMM (*n* = 107). They reported severe pain and disability for more than a decade and had failed a multitude of therapies. Common diagnoses included degenerative disc disease, spondylosis, stenosis, and scoliosis—yet not to a degree amenable to surgery. The six-month primary end point compared responder rates, defined by a 50% reduction in pain. Hierarchical analyses of seven secondary end points were performed in the following order: composite responder rate (numerical rating scale [NRS] or Oswestry Disability Index [ODI]), NRS, ODI, Pain Catastrophizing Scale responder rate, Patient Global Impression of Change (PGIC) responder rate, and Patient-Reported Outcome Measure Information System-29 in pain interference and physical function.

**Results:**

Intention-to-treat analysis showed a significant difference in pain responders on NRS between SCS (72.6%) and CMM (7.1%) arms (*p* < 0.0001). Of note, 85.2% of those who received six months of therapy responded on NRS compared with 6.2% of those with CMM (*p* < 0.0001). All secondary end points indicated the superiority of burst therapy over CMM. A composite measure on function or pain relief showed 91% of subjects with SCS improved, compared with 16% of subjects with CMM. A substantial improvement of 30 points was observed on ODI compared with a <one-point change in the CMM arm. Three serious and 14 non–serious device- or procedure-related events were reported.

**Conclusions:**

This study found substantial improvement at six months in back pain, back pain-related disability, pain-related emotional suffering, PGIC, pain interference, and physical function in a population with severe, debilitating back pain for more than a decade. These improvements were reported in conjunction with reduced opioid use, injection, and ablation therapy.

## INTRODUCTION

Low back pain (LBP) is a highly prevalent musculoskeletal problem and a leading cause of disability, affecting millions of people worldwide in all sociodemographic classes.^1^ Although most LBP episodes resolve within six weeks, nearly 35 million adults in the United States (US) (13.1%) experience chronic low back pain (CLBP) refractory to conservative algorithmic care.^2,3^ Prevalence of CLBP increases with age and is comparatively higher in women, current and former smokers, and obese individuals.^2,4^

Refractory axial CLBP can be caused by a variety of factors, including spinal stenosis, spondylosis, spondylolisthesis, facet joint disease, disc herniation, discogenic pain including degenerative disc disease, other structural deformities, scoliosis, and combinations of these conditions. Most patients presenting for treatment have >one pain generator, making a singular unifying diagnosis difficult and uncertain in many cases.^5^ Intervertebral discs, vertebrae and associated joints, soft tissues, and neurogenic vasculature compression can alone or in combination contribute to the painful condition, which may vary over time. A subset of these patients show multilevel imaging changes with no definitive causation of the symptoms. Although the exact etiology of the pain can be elusive, these patients are still experiencing substantial and disabling pain.^5^ The result is an underserved population of patients with CLBP with multiple potentials and still unclear underlying etiologies for pain, yet whose pathology is not correctable with surgery.^6–9^

These patients are typically treated conservatively, with physiotherapy, chiropractic care, massage or acupuncture, oral analgesics, and image-guided injections.^10–13^ Even when performed in a cautious, algorithmic manner, injections are rarely a long-term solution. For many of these patients, the conservative care approach is repeated in an ongoing cycle. The algorithm may progress in a stepwise fashion in order of increasing invasiveness, including repeated imaging and specialist visits with further attempts at radiofrequency ablation, decompression, and spine surgery for those with a clear anatomical target. This treatment continuum represents the best current practice of exposing the patient to the least invasive, least expensive treatment for improvement and typically places implantable neuromodulation technologies as a therapy of late or last resort. With prolonged unrelieved back pain, a patient’s physical condition deteriorates over time, with progressive impairment of function and with psychologic comorbidities such as depression and anxiety. Optimal timing of neuromodulation remains unclear, with older literature suggesting best outcomes were seen in patients with only a few years or less of chronic pain. Recent real-world evidence suggests that once the patient’s chronic pain is refractory, prolonged delay of neuromodulation beyond two years leads to increased costs without improving outcomes, signifying an optimal time to implement a more definitive treatment in those with established, refractory chronic pain.^14,15^

Spinal cord stimulation (SCS) has been used for decades to treat chronic back and leg pain, particularly in patients with persistent spinal pain syndrome (PSPS) type 1.^4,16^ Passive recharge burst SCS (B-SCS) is a unique stimulation design characterized by a five-pulse train with an internal frequency of 500 Hz delivered at 40 Hz, with a 1-millisecond pulse width.^17,18^ Charge accumulates during the intraburst phase, and after the burst packet, there is a period of passive discharge of energy. The accumulated charge gradually dissipates over time. Several randomized controlled trials have indicated the superiority of this waveform to conventional tonic SCS, including a recent meta-analysis.^19–22^ It uniquely mimics neuronal burst firing patterns in the nervous system and has been shown to modulate the affective, attentional components of pain processing in addition to the nociceptive components.^18^ A two-year study recently showed that B-SCS can alleviate pain intensity and pain-related emotional distress and improve physical function and health-related quality of life.^23^ Adults with self-reported CLBP lasting longer than three months are at risk of developing depression or anxiety disorders.^24^ These psychologic factors affect the course of the condition and an individual’s response to treatment.^24^ Importantly, B-SCS appears to be as effective in a population with chronic pain with high psychologic distress as in those without distress.^25^ Biopsychosocial dimension factors contribute to patient disability, creating an extremely expensive, impactful problem for patients, healthcare systems, and societal infrastructure.^26^

Primary and secondary results presented here evaluate pain relief, pain-related physical function, and disability, psychologic catastrophizing, and patient satisfaction. Furthermore, the use of pain medications and interventional treatments in each arm is reported.

### Study Objective

The Dorsal spInal cord STImulatioN vs mediCal management for the Treatment of low back pain (DISTINCT) study is a multicentered, prospective randomized controlled trial that evaluated the efficacy of SCS compared with that of conventional medical management (CMM) in improving pain and back pain-related physical function in patients with chronic, refractory axial low back pain (PSPS type 1), who had not undergone lumbar surgery and for whom surgery was not an option.

## MATERIALS AND METHODS

### Subject Population

All study documents received institutional review board approval before subject enrollment. The study is registered on ClinicalTrials.gov (NCT04479787). Consent was obtained from all potential subjects before enrollment. The study is conducted in accordance with the US Code of Federal Regulations and the World Medical Association Declaration of Helsinki.

Magnetic resonance imaging and/or computed tomography images of the spine obtained within 12 months were reviewed by an independent orthopedic spine surgeon before enrollment and randomization to confirm lack of an identifiable pathology that could effectively be treated with surgery. Back pain was the primary complaint; leg pain also could be present, but it could not exceed back pain in severity. Subjects reported moderate-to-severe back pain-related disability. All inclusion and exclusion criteria are listed in Table 1 and Table 2, respectively. The patients had exhausted numerous types of conservative care that were documented, including physiotherapy, medications including opioids, injections, radiofrequency ablations, chiropractic care, and massage, often on a repeated basis (Table 3).

Subjects were randomized in a 3:2 ratio to either the B-SCS arm or the CMM arm, stratified by site. This was to account for those subjects randomized to B-SCS who did not achieve ≥50% pain relief during the temporary B-SCS trial period failures and were ineligible to move to a permanent implant. All assignments were allocated using an electronic data capture system. The subjects, site personnel, and some sponsor personnel were aware of the treatment assignment. Statisticians did not have access to any data that combined outcomes with treatment assignment before performing the primary end-point analysis of the randomized cohort. Device safety in this population was monitored using an independent clinical events committee.

### CMM Treatments

Subjects randomized to the CMM arm received supervised medical care, including physical modalities, medication optimization, and interventional therapies depending on the diagnosis and as decided by the investigator. Medication optimization could include using nonsteroidal antiinflammatories, anticonvulsants, muscle relaxants, opioids, and other analgesics as appropriate. Supervised noninterventional therapy could include physical therapy (including back school), chiropractic care, cognitive behavioral therapy, massage, and acupuncture. Interventional therapies, such as injections and radiofrequency therapy, also were allowed.

### SCS Intervention

BurstDR^™^-capable implantable pulse generators (IPGs), along with relevant leads and accessories, were used in accordance with Food and Drug Administration-approved instructions for use. All subjects randomized to B-SCS underwent a four-to-seven–day trial period, with lead location per the physician’s customary practice for treating low back pain. B-SCS stimulation parameters were configured using the clinician programmer and delivered using an external pulse generator. Subjects proceeded to an IPG implant after a successful temporary SCS trial period, defined as ≥50% reduction in back pain measured by a numeric rating scale (NRS). Percutaneous or paddle leads were used at the implant depending on the surgeon’s preference. A choice of recharge-free or rechargeable IPG was used (Proclaim or Prodigy, Abbott, Plano, TX).

### Follow-Up

**Figure 1.**
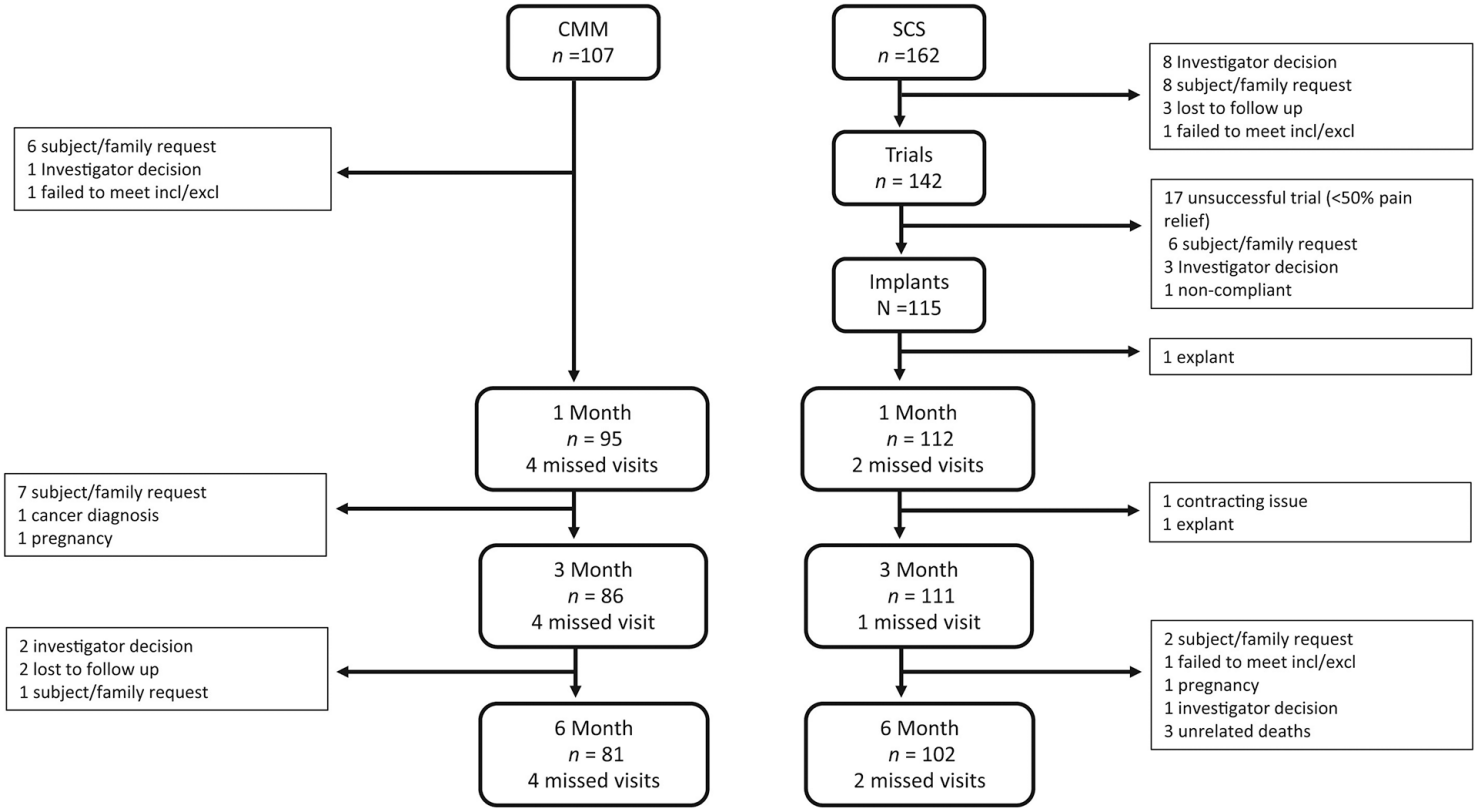
Consort diagram for 269 subjects randomized and disposition through the six-month follow-up visit. One patient was enrolled, but the patient was never randomized. Patient withdrawal by sponsor request was due to contract and budgeting difficulties. Patients who failed to meet the inclusion and exclusion criteria after randomization were withdrawn after the occurrence of a monitoring visit that uncovered medical conditions that should have excluded the patient. The patient excluded after three months was excluded before the six-month primary end point.

A CONSORT flow diagram is illustrated in Figure 1. Subjects were observed in clinic for required study visits at one, three, and six months. Future study in-clinic visits are planned at nine, 12, 18, and 24 months and optional in-clinic visits or phone calls at 15 and 21 months. A provision was allowed for remote telemedicine as provided by each study center if an in-clinic visit could not occur owing to reasons such as COVID-19 or logistic restrictions. For subjects experiencing difficulty accessing in-clinic care, a telehealth appointment with remote programming of SCS devices could occur as needed (Neurosphere Virtual Clinic, Plano, TX). After the primary end point was assessed at six months, subjects could elect to cross over to the other treatment arm. Subjects who crossed over to the SCS treatment arm followed the standard trial and implant procedures.

### Statistical Analysis

Enrollment occurred in two phases: primary analysis cohort (*n* = 200) and continued access phase (*n* = 70). The primary analysis cohort of 200 randomized subjects was statistically powered (>90%) to identify improvements in pain intensity and back pain-related function in the SCS arm superior to those in the CMM arm at six months. Assumptions included in this sample size analysis were a 25% difference in responder rates, a 25% attrition rate, and a two-sided ɑ of 0.05. An interim analysis of 70% of the primary cohort was prespecified to confirm the study’s statistical power for 200 subjects. The enrollment of another 70 subjects further adds to the body of evidence supporting B-SCS effectiveness in this patient population.

The study end points incorporated outcome measures and associated clinically meaningful improvements based on the Initiative on Methods, Measurement, and Pain Assessment in Clinical Trials (IMMPACT) guidelines.^27^ Data collected included measures of pain (NRS), back pain-related physical function (Oswestry Disability Index [ODI]), pain-related emotional distress (Pain Catastrophizing Scale [PCS]), and quality of life (Patient-Reported Outcome Measure Information System [PROMIS] domains and two PROMIS Cognitive Function Abilities items [PROMIS−29+2]). In addition to pain-related medicine usage and healthcare utilization, a seven-point Likert scale for Patient Global Impression of Change [PGIC] and a four-point Likert scale for satisfaction were collected.

The primary end point evaluated the difference in responder rates between the SCS and CMM randomized groups at six months. Responders were defined by a ≥50% reduction in LBP, measured by NRS. An intention-to-treat (ITT) analysis included subjects who failed the SCS trial period as nonresponders. A per-treatment evaluation (PTE) analysis also was performed for all subjects with complete six-month data. Hypothesis testing for superiority used a two-sided Z-test with unpooled variance at the significance level of 0.05.

The seven secondary end points were tested in hierarchical order to control for type I error inflation (Table 4). Responder analyses used literature-supported clinical meaningful differences (NRS, ODI, PCS, and PGIC). A composite measure of therapy effectiveness is the first secondary end point to jointly assess improvements in pain and function between the two treatment groups. Composite outcome measures better reflect and predict a patient’s impression of change after therapy.^28–30^ Pain and function are interrelated outcomes, and improvements on either are recognized as effective treatment by both physicians and patients.^21^ Responders were defined by a meaningful improvement on pain intensity (≥50% reduction on NRS) or a meaningful improvement in back pain-related function (≥13 percentage points decrease on ODI or score ≤20).^31^ Outcomes on PCS have been evaluated to set scores expected in normal populations without chronic pain and changes in scores that are clinically meaningful to patients. A score of 13.87 is representative of a normal, healthy population.^32^ A total score of ≥30 indicates a patient is clinically catastrophizing. A 38% to 44% reduction in score represents a noticeable improvement for the patient.^33^ PCS responders were defined as subjects who are either clinically catastrophizing on PCS at baseline (PCS score ≥30) and report a score of <30 at six months follow-up or report a 40% decrease in score at six months follow-up compared with baseline, regardless of their baseline scores. A PGIC responder is defined as a patient who reported “better” or “great deal better” at six months.

All end points are summarized at baseline and follow-up visits up to the primary end point. Continuous variables are presented as means, SDs, and 95% CIs of the mean. Categorial variables are summarized as percentages and where applicable, with exact 95% Clopper–Pearson confidence intervals.

All data analysis was performed using SAS version 9.4 (SAS Institute Inc, Cary, NC).

## RESULTS

### Patient Disposition

The study enrolled 270 subjects between September 2020 and September 2021. A total of 107 subjects were randomized to the CMM arm and 162 to the B-SCS arm. The complete disposition of subjects up to the six-month primary end point is shown in Figure 1. Demographics and clinical characteristics at baseline are listed in Table 3. The mean ± SD age of the study population at enrollment was 58 ± 13 years (30% <50 years). Subjects had experienced chronic pain for an average of 12.3 ± 11.3 years (50% >10 years) and over that time had been diagnosed with an average of three potential LBP generators, ranging from one to eight. Most had a broad diagnosis of CLBP (59%), and 80% carried additional spinal diagnoses that were felt to contribute to the patient’s CLBP. Individuals in this population experienced an average of 3.5 treatments and failed to achieve lasting pain relief from noninvasive to interventional options such as injections and radiofrequency therapies. Moreover, 94% had tried a combination of physical therapy, massage, and chiropractic therapy (Table 3); 86% (*n* = 233) received an average of 4.9 injections, and 41% (*n* = 110) underwent an average of 2.9 ablation procedures. In addition to these treatments, patients reported taking antiinflammatory (46%), opioid (42%), and/or anticonvulsant (32%) medications; 39% reported taking antidepression or antianxiety medication, and 25% were using sleep medication.

### Six-Month Primary End Point

**Figure 2.**
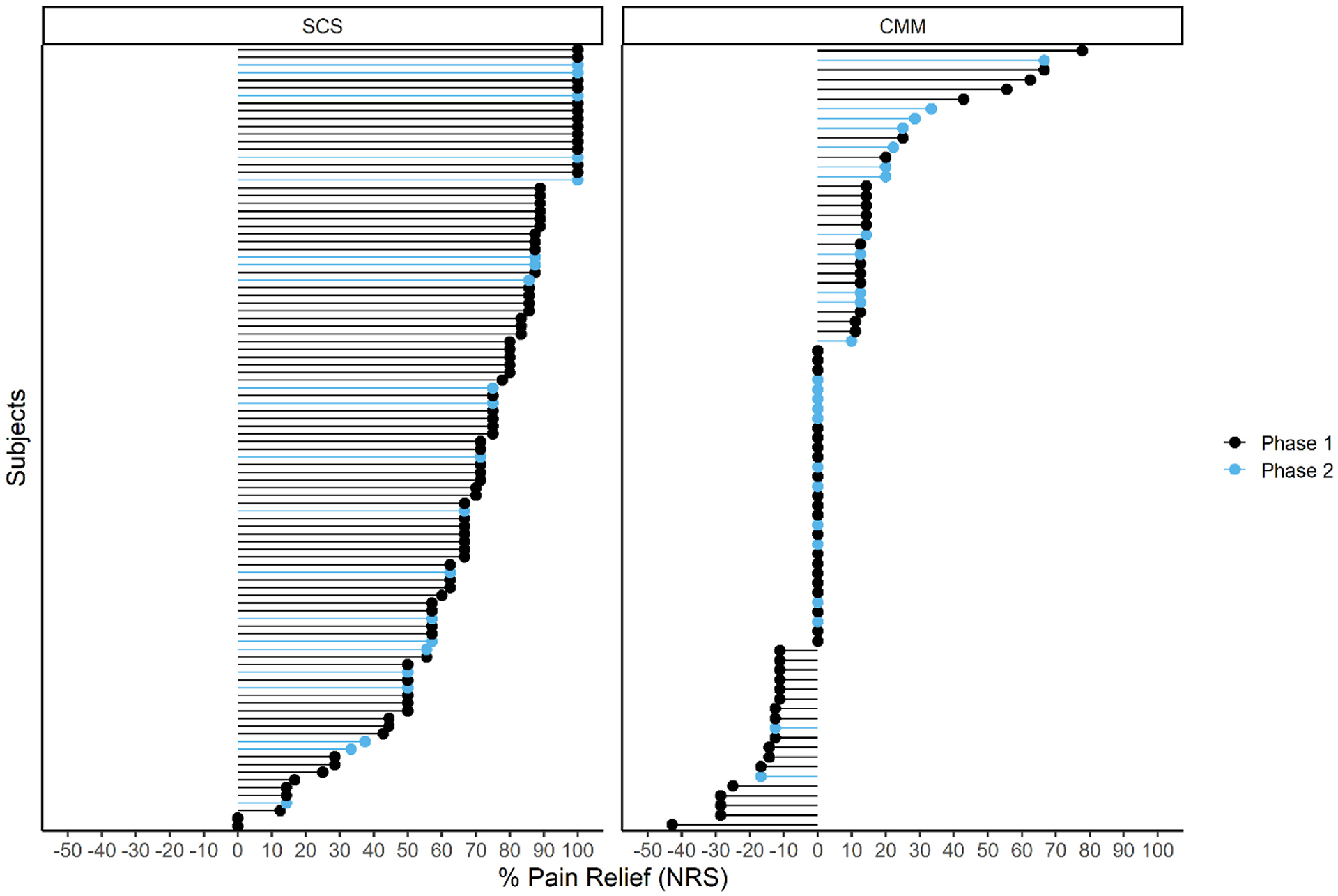
Individual pain relief for all subjects with six-month data. Phase 1 represents data from the first 200 randomized subjects for end-point analysis; phase 2 (blue) represents the next 69 randomized with outcome data.

The primary end point compared the proportion of responders (≥50% reduction on NRS) between B-SCS and CMM in the first 200 enrolled subjects. An ITT analysis that included the patients who failed the temporary B-SCS trial period as nonresponders revealed a 72.6% B-SCS responder rate (95% CI: 62.5%–81.3%) compared with 7.1% (95% CI: 2.0%–17.3%) in subjects randomized to CMM (*p* < 0.0001). A similar analysis of the 269 subjects was consistent (Table 4), reporting a 73.1% response rate after B-SCS therapy (95% CI: 64.2%–80.8%) and 6.2% after CMM (95% CI: 2.0%–13.8%). An individual NRS pain relief diagram is presented in Figure 2.

### Six-Month Secondary End Points

**Figure 3.**
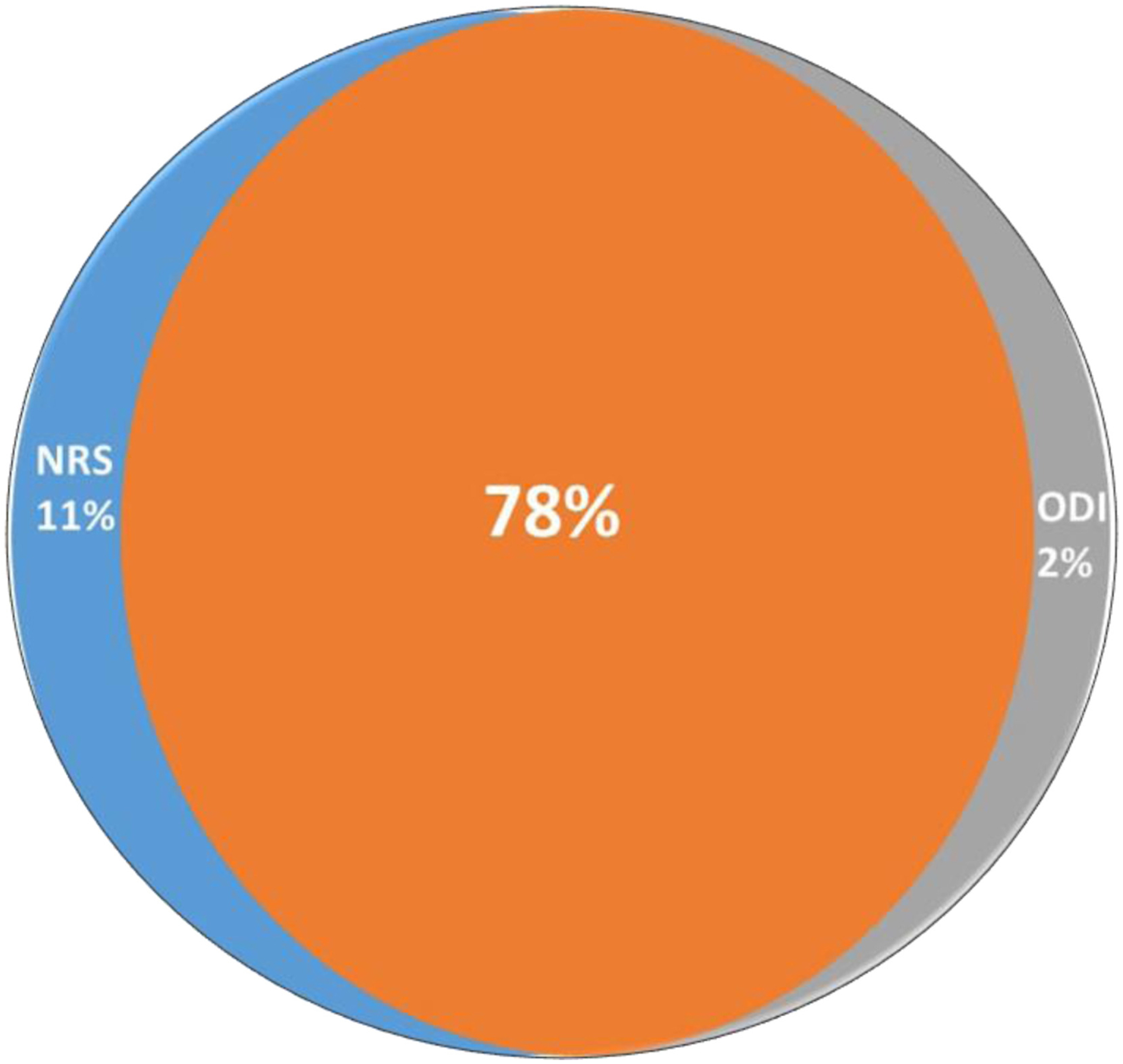
A composite end point evaluated pain relief (NRS) or functional improvement (ODI). Venn diagram shows 78% of subjects with SCS reported meaningful improvements on both. The end-point analysis reported a 91% responded rate after six months of passive recharge burst therapy.

**Figure 4.**
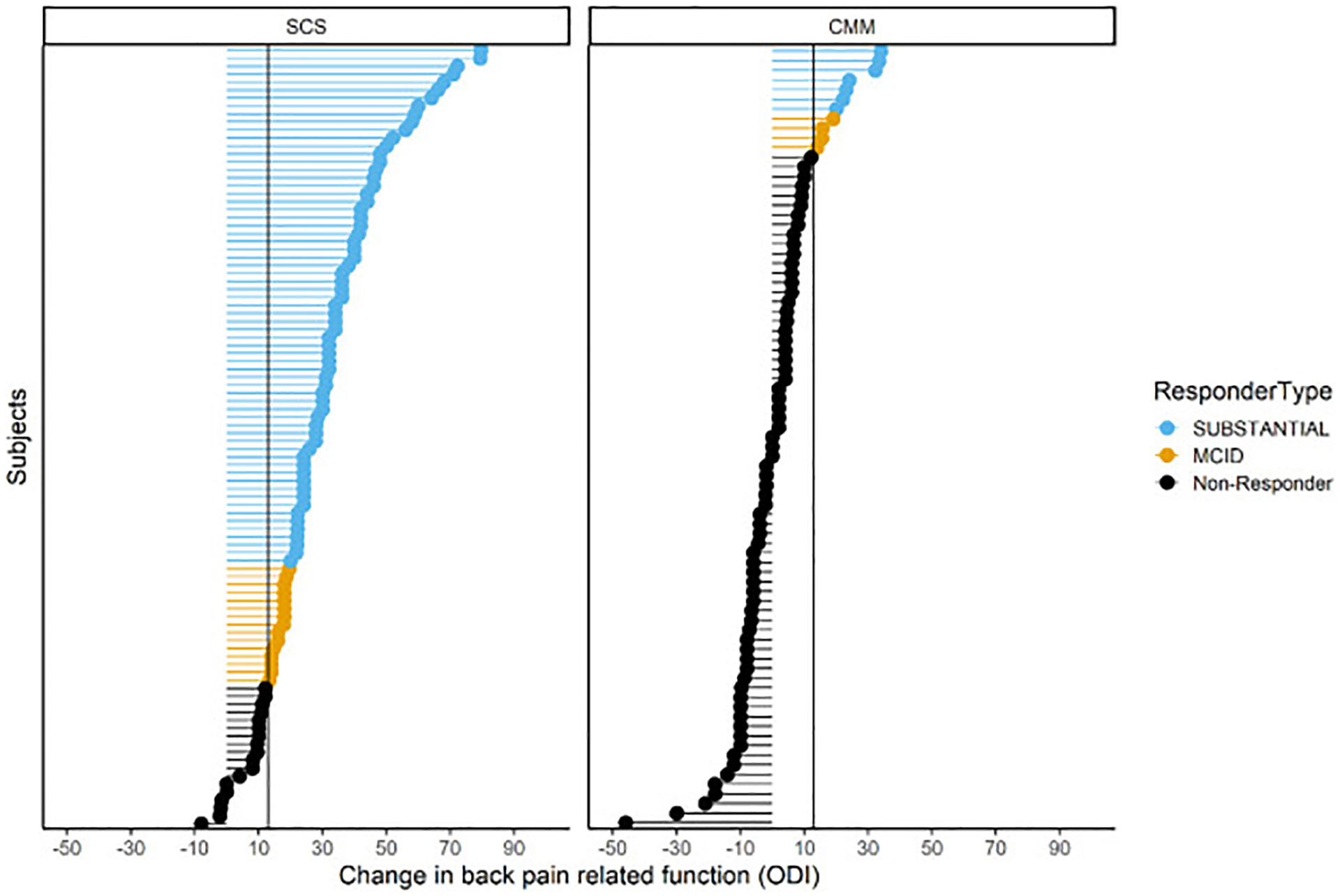
Improvements on ODI are shown for all subjects with six-month data. Blue represents a substantial improvement defined by ≥20-point improvement. MCID is defined as a 13-point improvement.^34^

All secondary end points were hypothesis tested on the first 200 subjects randomized and described using the 269 cohort. The first secondary end point tested a composite measure of pain (NRS) or function (ODI) responder status (Table 4) in the first 200 randomized subjects. The B-SCS arm revealed a greater response rate than that of CMM (91.4% vs 19.6%; *p* < 0.0001; *n* = 200). An analysis of all 269 subjects was consistent and showed 91.2% of subjects with SCS (93/102) responded on either measure compared with 16.0% of subjects in the CMM arm (13/81) (Table 4). Of the 93 responders, 80 (78%) responded on both NRS and ODI (Fig. 3); 11 subjects responded just on NRS and did not meet the threshold for minimal clinically important difference (MCID = 13) on ODI.^34^ The average ODI improvement reported by this subset was 7.4. Six subjects responded on ODI only. The average reduction in pain score in these subjects was 37.8%, above the accepted MCID of 30%.^34^ This supports a clinically meaningful improvement in pain but not reaching the 50% improvement classified as a substantial improvement by the IMMPACT guidelines. The patient data point for disability improvement is presented in Figure 4.

**Figure 5.**
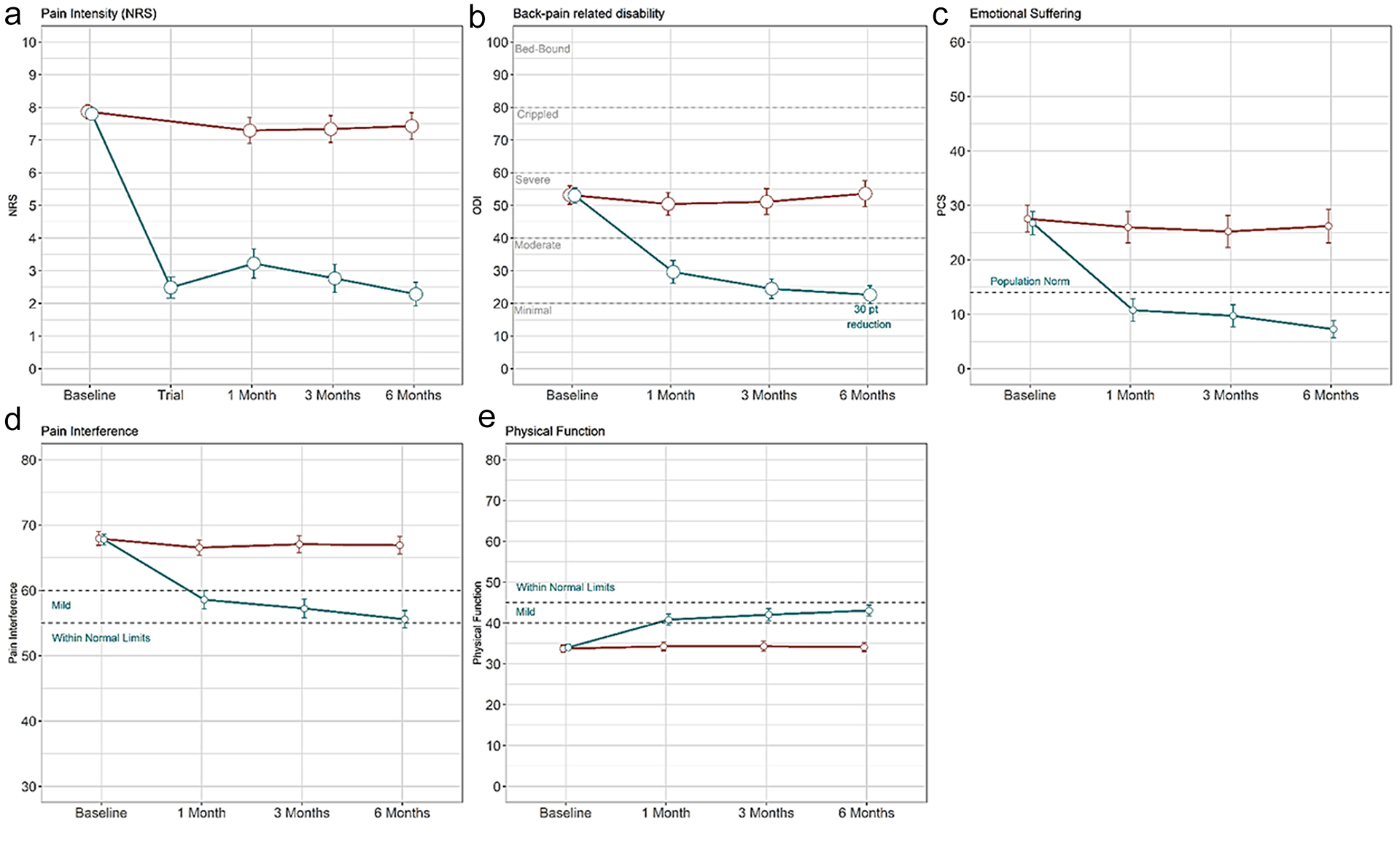
Line plots show all secondary end-point measures that show greater improvements on a continuous scale after SCS (blue) than with CMM (red). Panels a–e represent NRS, ODI, PCS, PROMIS-29 pain interference, and PROMIS-29 physical function instruments, respectively. Hypothesis testing was conducted for the secondary end-point analysis. All baseline-to-six-month change values are statistically significant (*p* < 0.05).

A PTE analysis at six months for NRS showed 85.2% responded in the B-SCS arm compared with 7.1% with CMM (*p* < 0.0001). Consistent pain relief metrics were reported in the 269 cohort (85.3% vs 6.2%; Fig. 1). Back pain in the B-SCS group was reduced to 2.3 ± 1.8, 5.1 points greater than in the CMM group (Fig. 5a). In the treatment arm, 61% of subjects (61/102) reported an NRS score of ≤2, a benchmark previously used to indicate remittance or resolution of chronic back pain;^35,36^ 18 of those subjects (18%) reported 100% percent pain relief.

A substantial ODI improvement of 30.6 points (95% CI: 26.2–35.1) was reported by the B-SCS group compared with 0.7 points (95% CI: −3.1 to 4.6) in the CMM arm (*p* < 0.0001). Consistent results were reported for all subjects in the 269 cohort with six-month follow-up data (Table 4). The treatment arm decreased from a score of 52.5 ± 13.8, indicating severe disability, at baseline to a moderate disability score of 22.6 ± 13.8 at six months (Fig. 5b). Similarly, subjects with CMM reported severe disability at baseline (53.2 ± 14.6) but remained severely disabled after six months of treatments (53.6 ± 18.1). Moreover, 83.7% of subjects with SCS (82/98) experienced an MCID of 13 points, and 66.3% reported a substantial improvement of ≥20 points (Fig. 4). This contrasted to 14.8% of subjects with CMM (12/81) reporting MCID, and 8.6% reporting substantial improvement.

A total of 88.2% of subjects with B-SCS (90/102) reported meaningful changes on the psychologic PCS instrument (95% CI: 80.4%–93.5%) compared with 23.5% of subjects with CMM (19/81) (95% CI: 14.8–34.3). The average score of the B-SCS arm improved from 27.6 to 7.3, below the average expected value for an adult population with nonchronic pain (Fig. 5c). Less than a two-point change was reported by the CMM group. A greater proportion of subjects who received B-SCS therapy noticed a definite or considerable improvement than those who received CMM therapy (75.5% [95% CI: 66.0–83.5] vs 2.5% [95% CI: 0.3%–8.6%] *p* < 0.0001).

On the PGIC scale, 75.5% of subjects with B-SCS (77/102) reported feeling better or a great deal better compared with 2.5% of subjects with CMM (2/81). The final two end points tested day-to-day activities and interference in those activities using pain interference (Fig. 5d) and physical function from the PROMIS-29 questionnaire (Fig. 5e). Both showed greater improvement in the B-SCS arm, 17.0% and 26.7%, respectively (*p* < 0.0001; Table 4). On physical function, subjects with B-SCS reported an average T-score improvement of 8.9 ± 7.0 from 34.1 ± 4.7 at baseline compared with a change of 0.3 ± 4.3 by subjects with CMM from baseline (33.6 ± 4.5). T-scores for pain interference were also greatly reduced in the subjects with B-SCS, improving from 68.0 ± 5.0 at baseline by 12.4 ± 8.2 compared with a change of 0.9 ± 6.4 in subjects with CMM from baseline (67.9 ± 5.3).

### Medication and Interventional Treatments

Of all subjects, 37% reported opioid use at baseline (96/269). The average morphine milligram equivalent (MME) reported by subjects randomized to B-SCS and CMM was 23.3 ± 19.3 and 24.9 ± 19.3, respectively; 51.2% (21/41) of subjects with SCS on opioids at baseline decreased use after six months of therapy. Within that group, 13 subjects stopped all opioid use. Moreover, 33% of subjects with CMM (9/27) with six-month follow-up decreased opioid use, and six stopped all opioid use. On average, subjects receiving B-SCS treatment reported a 45.3% decrease in MME at six months compared with baseline. The CMM group reported an 11.1% decrease in MME at six months compared with baseline.

During the six months of CMM treatment, 8.8% of patients (7/80) underwent physical therapy; 10.0% (8/80) received occupational therapy; 11.3% (9/80) received massage therapy; 5.0% (4/80) received chiropractic therapy, and 3.8% (3/80) used acupuncture.

Nine subjects with SCS reported ten injection treatments compared with 59 in 32 of subjects with CMM. Two ablation procedures occurred in the SCS group, whereas 16 were reported in the CMM group.

### Safety

Fourteen nonserious device- or procedure-related events were reported in the B-SCS arm (14/162, 8.6%). All events were known potential B-SCS complications and followed expected frequencies. Six were related to lead migrations (3.7%), and two were infections (1.2%). Two skin reactions were reported, two cases with pain at the site of the implant, one cerebrospinal fluid leakage, and one IPG migration. Three serious events were reported. Two were infections that required explant. One was postprocedural abdominal pain resolved with pain management without sequelae. Three deaths occurred in the B-SCS arm (pulmonary edema, digoxin toxicity, and bladder rupture), all nonrelated to the device or procedure. No serious events were observed in the CMM arm.

## DISCUSSION

The DISTINCT study compares the effectiveness of SCS with that of CMM in a population with chronic back pain without options for corrective surgery. Historically, these patients have often been represented within the populations enrolled in general chronic pain SCS studies; however, rigorously designed studies powered to evaluate SCS in this specific patient population are limited.^37^ To our knowledge, this is the first such study to evaluate passive recharge B-SCS in this population. Trialing and implanting physicians represented multiple specialties, including interventional pain, neurosurgery, and orthopedic spine surgery, reflective of the physicians typically treating this population. The study was statistically powered to test one primary and seven secondary end points. All were successful and show greater improvements in the B-SCS arm.

The primary end point supports substantial improvements in pain intensity. In an ITT analysis, 73.1% of subjects randomized to SCS responded with 50% greater pain relief compared with 6.2% randomized to CMM (95% CI: 54.3–76.75). An analysis of subjects receiving stimulation (PTE) at six-month follow-up showed 85% responded (95% CI: 76.1%–91.8%) compared with 6.2% of subjects with CMM (95% CI: 2.0%–13.8%).

Chronic refractory pain is a complex biopsychosocial disease, multiple dimensions of which contribute to patient disability, creating an extremely expensive and impactful problem for patients, healthcare systems, and societal infrastructure^1^. The secondary effectiveness end points of the DISTINCT study reflect the complex biopsychosocial nature of chronic pain, including pain, function, psychologic distress, and well-being. A profound improvement in ODI, measuring >30 points, is observed in the treatment arm compared with zero change in the CMM arm. This is more than twice the MCID (MCID = 13 points). Pain catastrophizing was greatly improved, suggesting that subjects with SCS ruminate less, magnify their pain less, and have fewer feelings of helplessness around their chronic pain condition. This is consistent with an overall psychologic improvement. The average PCS score of 7.3 after SCS treatment reflects a normal population without chronic pain, falling below the published mean of 13.87.^32^ That such improvement was observed in a population who had experienced severe pain and disability for more than a decade and had not responded to the typical standard of care treatment speaks to the significance of this study.

Single univariate outcome measures for pain intensity (VAS, NRS) should not be relied upon exclusively to evaluate therapy success; a multidimensional combination of outcomes provides a more meaningful measure of holistic response.^29,30,38^ An important component of the DISTINCT study design is a composite secondary end point that addresses the interplay of pain intensity and back pain-related function. Treatment success reported by 91% of subjects receiving passive recharge burst therapy is not confined to reducing pain intensity but also captures improving function. The degree of pain relief does not always directly correlate with improvements in disability, and small changes in one measure can be observed with more significant changes in other measures. Measures such as these better capture a patient’s preference for therapy, satisfaction, and improvements in quality of life. Although previous neuromodulation studies have traditionally focused on the composite end points of safety and therapy effectiveness, they have yet to embrace the wider variety of validated clinical outcome measures currently available such as PCS and PROMIS scales^17^. Combining multiple outcome measures, which incorporates the relationship between domains, moves us toward more clinically relevant effectiveness measures^14–16^. Pain and function are interrelated outcomes, and improvements in either are recognized as an effective treatment by both physicians and patients^21^. The improvements noted in our composite outcomes reflect substantial holistic improvement in patients with long-standing chronic back pain.

### Limitations

The DISTINCT study was designed to follow best medical practice in both randomized arms. Currently, this prevents blinding of study subjects, physicians, or study site personnel to the treatment assignment. To mitigate potential bias, independent experts were used at different decision points in the study. Appropriate subject enrollment, specifically to the lack of spine surgery indications, was independently verified by an orthopedic spine surgeon, who functioned as an independent medical monitor before randomization. A predefined interim analysis was conducted by an independent statistician to verify the appropriate sample size and statistical power necessary for primary and secondary end-point analysis. An ITT analysis was performed as part of the primary end point, which supported a substantial, statistically significant difference in pain reduction due to SCS compared with CMM alone. The protocol did not standardize or limit the noninterventional or interventional therapies that could be prescribed during the study. This allowed individualized and optimized care in the CMM arm, providing the opportunity for a new treatment algorithm experience, supporting clinical equipoise, and indicated by improvements observed in a limited number of subjects randomized to CMM after six months. All meaningful patient-reported outcomes tested showed greater improvement in response to passive recharge burst therapy than to CMM. In addition, medications and interventional therapy usage were reduced. A full healthcare utilization and cost-effectiveness analysis of these data is required for appropriate interpretation. These initial results six months after implant are extremely compelling; patients will have one-year, 18-month, and two-year follow-ups to provide evidence for long-term improvements. Finally, every clinical study is affected by the placebo effect. To reduce the placebo effect, the research end point was set at six months rather than three months. Furthermore, the degree of the response indicates a therapeutic influence. The study is designed to observe subjects to 24 months; thus, this long-term follow-up may mitigate concerns about a placebo effect.

## CONCLUSIONS

The DISTINCT study results indicate that passive recharge B-SCS is superior to CMM for patients experiencing severe, debilitating low back pain that cannot be addressed through corrective surgical intervention. Primary and secondary end points show dramatic improvements in pain, function, pain-related emotional distress, day-to-day pain interference, and a greater PGIC. Greater improvements with passive recharge B-SCS therapy are noted, using fewer injection and RF ablation procedures and accompanied by reductions in opioid use. Strengths of the study included the combination of orthopedic spine surgery, neurosurgery, and interventional pain investigator sites, and the inclusion of patients with long-standing, refractory axial spine without corrective surgical indications.

## Figures and Tables

**Table 1.** Inclusion Criteria.

1. Patient must be willing and able to provide written informed consent before any clinical investigation-related procedure
2. Age ≥18 y
3. Patient has chronic (≥6 mo), refractory axial low back pain with a neuropathic component and is not a candidate for spine surgery
4. Patient has back pain for ≥6 mo inadequately responsive to supervised conservative care
5. Patient has not had spine surgery for back or leg pain
6. Patient is a candidate for SCS
7. Low back pain ≥6 on NRS
8. ODI score of ≥30%
9. Willing and able to comply with the instructions for use, operate the study device, and comply with this clinical investigation plan

**Table 2.** Exclusion Criteria.

1. Pathology seen on imaging tests obtained within the past 12 mo that is clearly identified and is likely the cause of the CLBP, that can be addressed with surgery
2. Primary symptom of leg pain, or leg pain is greater than back pain
3. Back pain is due to any of the following: spinal instability defined as >2 mm translation on radiographic imaging visceral causes (eg, endometriosis or fibroids) vascular causes (eg, aortic aneurysm) spinal infection (eg, osteomyelitis) inflammation or damage to the spinal cord (eg, arachnoiditis or syringomyelia) tumor or spinal metastases
4. Has widespread pain (eg, fibromyalgia) or pain in other area(s), not intended to be treated in this study (eg, neck pain, shoulder pain)
5. Patient has seronegative spondyloarthropathy (eg, rheumatoid, lupus, psoriatic)
6. Neurologic deficit (eg, foot drop)
7. Previous lumbar spine surgery or sacroiliac joint fusion
8. Patient has used a morphine equivalent daily dose of >50 MME in the last 30 d
9. Patient is bedbound
10. Patients with regular intake of systemic steroids (except inhaled steroids used to treat asthma)
11. Imaging (MRI, CT, x-ray) findings within the last 12 mo that contraindicate lead placement
12. Known allergic reaction to implanted materials
13. Severe scoliotic deformity (>11° in thoracic or lumbar spine)
14. Patient has a history of or existing intrathecal drug pump
15. Patient has previous experience with neuromodulation devices, including a failed trial
16. BMI >40
17. Patient is enrolled, or intends to participate, in another clinical drug and/or device study or registry that may interfere with the results of this study, as determined by Abbott personnel
18. Presence of other anatomic or comorbid conditions, or other medical, social, or psychologic conditions that, in the investigator’s opinion, could limit the subject’s ability to participate in the clinical investigation or to comply with follow-up requirements of the clinical investigation results
19. Failed psychologic evaluation
20. Suspicion or evidence of untreated mental illness, substance abuse, or drug-seeking behavior
21. Patient revealed 2 or more Waddell’s signs of nonorganic behavior
22. Patient is in current litigation for back pain/injury, or is currently receiving worker’s compensation
23. Pregnant or nursing subjects and those who plan pregnancy during the clinical investigation follow-up period Women of child-bearing potential must have a negative pregnancy test done within 7 d before enrollment/baseline visit per site standard test

MRI, magnetic resonance imaging; CT, computed tomography.

**Table 3.** Baseline Demographics and Characteristics for All Randomized Subjects.

Demographics	SCS (*N* = 162)	CMM (*N* = 107)	Total (*N* = 269)
Age (y)			
Mean ± SD (*n*)	58.1 ± 13.0 (162)	59.1 ± 12.4 (103)	58.5 ± 12.8 (265)
Median (Q1, Q3)	59.0 (49.0, 67.0)	58.0 (50.0, 69.0)	58.0 (50.0, 68.0)
Sex, *n* (%)			
Female	59.3% (96/162)	50.5% (52/103)	55.8% (148/265)
Male	40.7% (66/162)	49.5% (51/103)	44.2% (117/265)
Race, *n* (%)			
White	83.3% (135/162)	84.5% (87/103)	83.8% (222/265)
Black or African American	8.0% (13/162)	2.9% (3/103)	6.0% (16/265)
Asian	6.2% (10/162)	9.7% (10/103)	7.5% (20/265)
American Indian or Alaska Native	0.6% (1/162)	1.9% (2/103)	1.1% (3/265)
Declined	1.2% (2/162)	1.9% (2/103)	1.5% (4/265)
Native Hawaiian or Other Pacific Islander	0.6% (1/162)	0.0% (0/103)	0.4% (1/265)
Pain NRS			
Lower Back			
Mean ± SD (*n*)	7.8 ± 1.2 (162)	7.9 ± 1.1 (107)	7.8 ± 1.2 (269)
Median (Q1, Q3)	8.0 (7.0, 9.0)	8.0 (7.0, 9.0)	8.0 (7.0, 9.0)
Right leg			
Mean ± SD (*n*)	2.9 ± 2.6 (140)	3.1 ± 2.8 (95)	3.0 ± 2.7 (235)
Median (Q1, Q3)	3.0 (0.0, 5.0)	3.0 (0.0, 6.0)	3.0 (0.0, 5.0)
Left leg			
Mean ± SD (*n*)	3.0 ± 2.8 (143)	3.0 ± 2.8 (94)	3.0 ± 2.8 (237)
Median (Q1, Q3)	3.0 (0.0, 5.0)	3.0 (0.0, 5.0)	3.0 (0.0, 5.0)
ODI			
Mean ± SD (*n*)	52.5 ± 13.8 (115)	53.2 ± 14.6 (107)	53.9 ± 14.2 (222)
Median (Q1, Q3)	53.3 (40.0, 62.0)	52.0 (42.2, 64.0)	52.0 (41.0, 63.0)
Pain distribution			
Back pain only	29.0% (47/162)	30.8% (33/107)	29.7% (80/269)
Back with unilateral leg pain	31.5% (51/162)	22.4% (24/107)	27.9% (75/269)
Back with bilateral leg pain	39.5% (64/162)	46.7% (50/107)	42.4% (114/269)
Duration of subject’s pain on subject’s life (y)			
Mean ± SD (*n*)	11.85 ± 10.58 (162)	13.06 ± 12.44 (103)	12.32 ± 11.33 (265)
Median (Q1, Q3)	10.00 (4.00, 15.00)	10.00 (4.00, 16.00)	10.00 (4.00, 15.00)
Pain diagnosis*			
Chronic, nonspecific, low back pain	60.5% (98/162)	61.2% (63/103)	60.8% (161/265)
Discogenic pain	6.8% (11/162)	7.8% (8/103)	7.2% (19/265)
Degenerative disc disease	30.9% (50/162)	36.9% (38/103)	33.2% (88/265)
Lumbar disc herniation	4.3% (7/162)	3.9% (4/103)	4.2% (11/265)
Lumbar facet arthropathy	21.6% (35/162)	25.2% (26/103)	23.0% (61/265)
Lumbar radiculopathy	34.6% (56/162)	43.7% (45/103)	38.1% (101/265)
Lumbar spinal stenosis	22.2% (36/162)	23.3% (24/103)	22.6% (60/265)
Lumbar spondylosis	48.1% (78/162)	58.3% (60/103)	52.1% (138/265)
Mechanical low back pain	7.4% (12/162)	3.9% (4/103)	6.0% (16/265)
Spondylolisthesis	6.2% (10/162)	4.9% (5/103)	5.7% (15/265)
Scoliosis	3.7% (6/162)	3.9% (4/103)	3.8% (10/265)
Other	7.4% (12/162)	15.5% (16/103)	10.6% (28/265)
Treatment for current condition*			
Physical Therapy	96.1% (148/154)	99.0% (98/99)	97.2% (246/253)
Occupational Therapy	13.0% (20/154)	16.2% (16/99)	14.2% (36/253)
Massage Therapy	38.3% (59/154)	39.4% (39/99)	38.7% (98/253)
Chiropractic Therapy	54.5% (84/154)	50.5% (50/99)	53.0% (134/253)
Acupuncture	33.8% (52/154)	22.2% (22/99)	29.2% (74/253)
Subject undergone any injections or interventions to treat their low back pain*			
Injection	97.4% (148/152)	91.4% (85/93)	95.1% (233/245)
Radiofrequency ablation/rhizotomy	42.1% (64/152)	49.5% (46/93)	44.9% (110/245)
Other	18.4% (28/152)	10.8% (10/93)	15.5% (38/245)

* Patients may report more than 1 treatment condition.

**Table 4.** Secondary End-Point Outcomes at Six Months.

Outcome	*N* = 200				*N* = 269		
	SCS	CMM	Difference [95% CI]^†^	*p* Value	SCS	CMM	Difference [95% CI]^†^
#1. Composite Responder	91.4% (74/81) [83.0%, 96.5%]	19.6% (11/56) [10.2%, 32.4%]	71.7% [59.6%, 83.8%]	< 0.0001^‡^	91.2% (93/102) [83.9%, 95.9%]	16.0% (13/81) [8.8%, 25.9%]	75.1% [65.4%, 84.8%]
Rate Percent [95% CI]*	[83.0%, 96.5%]				[83.9%, 95.9%]		
#2. NRS % change Mean ± SD (n) [95% CI]^†^	69.7 ± 25.0 (81) [64.2, 75.2]	3.6 ± 22.7 (56) [−2.5, 9.7]	66.1 [57.9, 74.2]	<0.0001^§^	69.7 ± 24.9 (102) [64.8, 74.6]	5.6 ± 21.3 (81) [0.9, 10.3]	64.1 [57.4, 70.9]
Mean ± SD (n) [95% CI]^†^	[64.2, 75.2]				[64.8, 74.6]		
#3. ODI Change	30.6 ± 19.8 (78)	0.7 ± 14.4 (56) [−3.1, 4.6]	29.9 [24.1, 35.8]	<0.0001^§^	29.4 ± 18.8 (98)	0.7 ± 13.0 (81) [−2.2, 3.5]	28.8 [24.0, 33.5]
Mean ± SD (*n*) [95% CI]^†^	[26.2, 35.1]				[25.7, 33.2]		
#4. PCS Responder	86.4% (70/81)	21.4% (12/56) [11.6%, 34.4%]	65.0% [51.9%, 78.1%]	< 0.0001^‡^	88.2% (90/102)	23.5% (19/81) [14.8%, 34.2%]	64.8% [53.6%, 75.9%]
Rate Percent [95% CI]*	[77.0%, 93.0%]				[80.4%, 93.8%]		
#5. PGIC Responder Rate	77.8% (63/81)	3.6% (2/56) [0.4%, 12.3%]	74.2% [63.9%, 84.5%]	< 0.0001^‡^	75.5% (77/102)	2.5% (2/81) [0.3%, 8.6%]	73.0% [64.0%, 82.0%]
Percent [95% CI]*	[67.2%, 86.3%]				[66.0%, 83.5%]		
#6. Pain Interference %	18.1 ± 11.4 (81)	1.1 ± 10.6 (56) [−1.8, 3.9]	17.0 [13.3, 20.8]	<0.0001^§^	17.9 ± 11.3 (102)	0.9 ± 9.3 (81) [−1.2, 3.0]	16.9 [13.9, 20.0]
Change Mean ± SD (*n*) [95% CI]^†^	[15.6, 20.6]				[15.6, 20.1]		
#7. Physical function %	28.0 ± 25.1 (81)	1.3 ± 13.1 (56) [−2.2, 4.8]	26.7 [20.2, 33.2]	<0.0001^§^	27.8 ± 24.4 (102)	1.4 ± 13.6 (81) [−1.6, 4.4]	26.3 [20.7, 32.0]
Change Mean ± SD (*n*) [95% CI]^†^	[22.5, 33.6]				[23.0, 32.6]		

*p* Value < 0.05 indicates end point is met.

* Exact Clopper-Pearson.

† By normal approximation.

‡ Two-sided Z-Test with unpooled variance.

§ Two-sample t-test.

## COMMENTS

The DISTINCT study does a tremendous service to the pain and medical community by adding support of SCS as a superior treatment to medication management for severe unrelenting pain. As the field advances with novel wave forms and paradigms, it was important to update the ever-important Process Trial with pragmatic approaches and studies in patients with complex pain. This study adds significantly to the body of evidence supporting spinal cord stimulation in patients for chronic back pain without previous surgery.

Peter Staats, MD, MBA

Frederick, MD, USA

The authors conducted an interesting randomized controlled trial to evaluate passive recharge burst versus conventional medical management in patients with chronic low back pain without previous spinal surgery (ie, patients with persistent spinal pain syndrome type I). These findings indicate that SCS should be considered earlier in the treatment continuum of chronic low back pain and not only serve as a last-resort treatment option when patients had already undergone surgical interventions. This study may serve as a potential starting point to initiate a revision of reimbursement criteria in the long term. Furthermore, the awareness has increased to incorporate composite measures of a holistic response as outcome measures in clinical trials. Within the field of neuromodulation, several definitions of holistic responses were previously implemented and recently summarized, together with a proposal on how to evolve in the future (Goudman L, Pilitsis JG, Russo M, et al. From pain intensity to a holistic composite measure for spinal cord stimulation outcomes. *Br J Anaesth*. 2023;131:e43–e48). In the current study, SCS seemed to outperform conventional management in a holistic response, whereby holistic responses will hopefully be embedded in most future trials of neuromodulation.

Lisa Goudman, MSc

Brussels, Belgium

I believe this is a study that requires a great deal of effort on the part of researchers. The authors should be congratulated on incorporating a much-needed interdisciplinary concept (cooperation of neurosurgeons, orthopedic surgeons, and pain physicians). The article assesses the effectiveness of passive recharge spinal cord stimulation (B-SCS) compared with conventional medical management (CMM) in a population of patients with long-standing chronic axial low back pain (CLBP) without any options for corrective surgery (PSPS type I). Such patients usually have multiple pain generators, often making diagnosis and treatment uncertain. The authors implement composite outcome measures that better reflect the patients’ perception of change, incorporate the relationship between pain and function domains, represent more clinically relevant measures of the SCS effectiveness, and provide a better measure of the holistic improvement/response in patients with CLBP. The results show that B-SCS is remarkably superior to CMM, with strong clinical implications. At six months, a >50% reduction in back pain (NRS) could be achieved in 85% of the SCS group vs 6.2% in the CMM. Even more impressively, with SCS, the ODI was reduced by 30.6 points, whereas with CMM, a minimal change of 0.7 points was observed. In the psychologic domain, 88.2% of the patients with B-SCS reported meaningful changes in the PCS compared with 23.5% of patients with CMM. In the B-SCS arm, 51% of patients decreased the use of opioids (45% decrease in MME) compared with a 33% reduction in patients with CMM (11% decrease in MME). In the B-SCS group, fewer injection treatments and fewer ablation procedures were undertaken than in the CMM group, ten vs 59, and two vs 16, respectively. B-SCS appears to be an effective way to complement our armory in neurostimulation/ neuromodulation for PSPS type I if no other surgical intervention is indicated. It should be considered useful in directly influencing not only pain scores but also function. I am looking forward to the two-year follow-ups.

Georgios Matis, MD, MSc, PhD

Cologne, Germany
